# Autophagy controls mesenchymal stem cell properties and senescence during bone aging

**DOI:** 10.1111/acel.12709

**Published:** 2017-12-06

**Authors:** Yang Ma, Meng Qi, Ying An, Liqiang Zhang, Rui Yang, Daniel H Doro, Wenjia Liu, Yan Jin

**Affiliations:** ^1^ State Key Laboratory of Military Stomatology & National Clinical Research Center for Oral Diseases & Shaanxi International Joint Research Center for Oral Diseases Center for Tissue Engineering School of Stomatology The Fourth Military Medical University Xi'an Shaanxi China; ^2^ Department of Craniofacial Development and Stem Cell Biology Dental Institute Kings College London London UK; ^3^ State Key Laboratory of Military Stomatology & National Clinical Research Center for Oral Diseases & Shaanxi International Joint Research Center for Oral Diseases Department of Periodontology School of Stomatology The Fourth Military Medical University Xi'an Shaanxi China; ^4^ Xi'an Institute of Tissue Engineering & Regenerative Medicine Xi'an Shaanxi China; ^5^ Department of Stomatology PLA Army General Hospital Beijing China

**Keywords:** adipogenesis, aging, autophagy, BMMSCs, osteogenesis, senile osteoporosis

## Abstract

Bone marrow‐derived mesenchymal stem cells (BMMSCs) exhibit degenerative changes, including imbalanced differentiation and reduced proliferation during aging, that contribute to age‐related bone loss. We demonstrate here that autophagy is significantly reduced in aged BMMSCs compared with young BMMSCs. The autophagy inhibitor 3‐methyladenine (3‐MA) could turn young BMMSCs into a relatively aged state by reducing their osteogenic differentiation and proliferation capacity and enhancing their adipogenic differentiation capacity. Accordingly, the autophagy activator rapamycin could restore the biological properties of aged BMMSCs by increasing osteogenic differentiation and proliferation capacity and decreasing adipogenic differentiation capacity. Possible underlying mechanisms were explored, and the analysis revealed that autophagy could affect reactive oxygen species and p53 levels, thus regulating biological properties of BMMSCs. In an in vivo study, we found that activation of autophagy restored bone loss in aged mice. In conclusion, our results suggest that autophagy plays a pivotal role in the aging of BMMSCs, and activation of autophagy could partially reverse this aging and may represent a potential therapeutic avenue to clinically treat age‐related bone loss.

## 1 INTRODUCTION

Senile osteoporosis is a systemic skeletal disease characterized by decreased bone mass and deterioration of bone micro‐architecture, leading to bone fragility and a high incidence of fractures (Raisz & Rodan, 2003). With catastrophic outcomes including pain, disability and mortality, osteoporosis and osteoporotic fractures have been major health problems among the aging population.

Mesenchymal stem cells (MSCs) are pluripotent cells that play crucial roles in tissue maintenance, repair and regeneration due to their self‐renewal and multilineage differentiation capacity. However, data suggest that beneficial functions of MSCs may become compromised with age; this is closely associated with age‐related loss of repair and regenerative capacity of different tissues (Wilson, Shehadeh, Yu, & Webster, 2010). Bone marrow‐derived mesenchymal stem cells (BMMSCs) decline in number with aging and show degenerative properties including reduced osteogenic differentiation capacity, increased adipogenic differentiation capacity and reduced proliferative ability; these are partially caused by bone aging (Wilson et al., 2010; Zhou et al., 2008). Different mechanisms of MSC senescence have been demonstrated including telomere shortening (Baxter et al., 2004), increased reactive oxygen species (ROS) (Stolzing & Scutt, 2006) and transcriptional control (Li et al., 2017). However, the complex molecular network is still largely unknown. Thus, a better understanding of the mechanisms of senescence in MSCs is required.

Autophagy is a process in which cellular components such as proteins and damaged mitochondria are engulfed by autophagosomes and delivered to lysosomes to be degraded and recycled in order to maintain cellular homeostasis (Mizushima, Levine, Cuervo, & Klionsky, 2008). Autophagy has been widely studied as a mechanism for anti‐aging effects and in alleviating age‐related diseases (Rubinsztein, Marino, & Kroemer, 2011). Recent studies have indicated that autophagy is required for maintaining the stemness and differentiation capacity of stem cells. It has been reported that basal autophagy is a crucial mechanism in the maintenance of the young state of satellite cells, and failure of autophagy causes cell senescence characterized by declines in number and function of satellite cells (Garcia‐Prat et al., 2016). Autophagy can protect BMMSCs from oxidative stress (Song, Song, & Tong, 2014), which indicates that autophagy plays a protective role in cell aging. Conversely, autophagy also has been proven to be a requirement for maintenance of replicative senescence of MSCs (Zheng et al., 2014). Therefore, whether and how autophagy regulates MSC aging remains unclear.

Recent evidence has shown that autophagy is a key regulator of bone metabolism (Onal et al., 2013). Here, we report that autophagy plays a role in the maintenance of BMMSCs during aging, and we demonstrate that regulation of autophagy can partially restore aged BMMSCs' properties and bone loss in mice via regulating ROS‐p53. These results suggest that bone aging may be partially orchestrated by autophagy of BMMSCs.

## 2 RESULTS

### 2.1 Bone mass declines during aging and BMMSCs display degenerative properties

**Figure 1:**
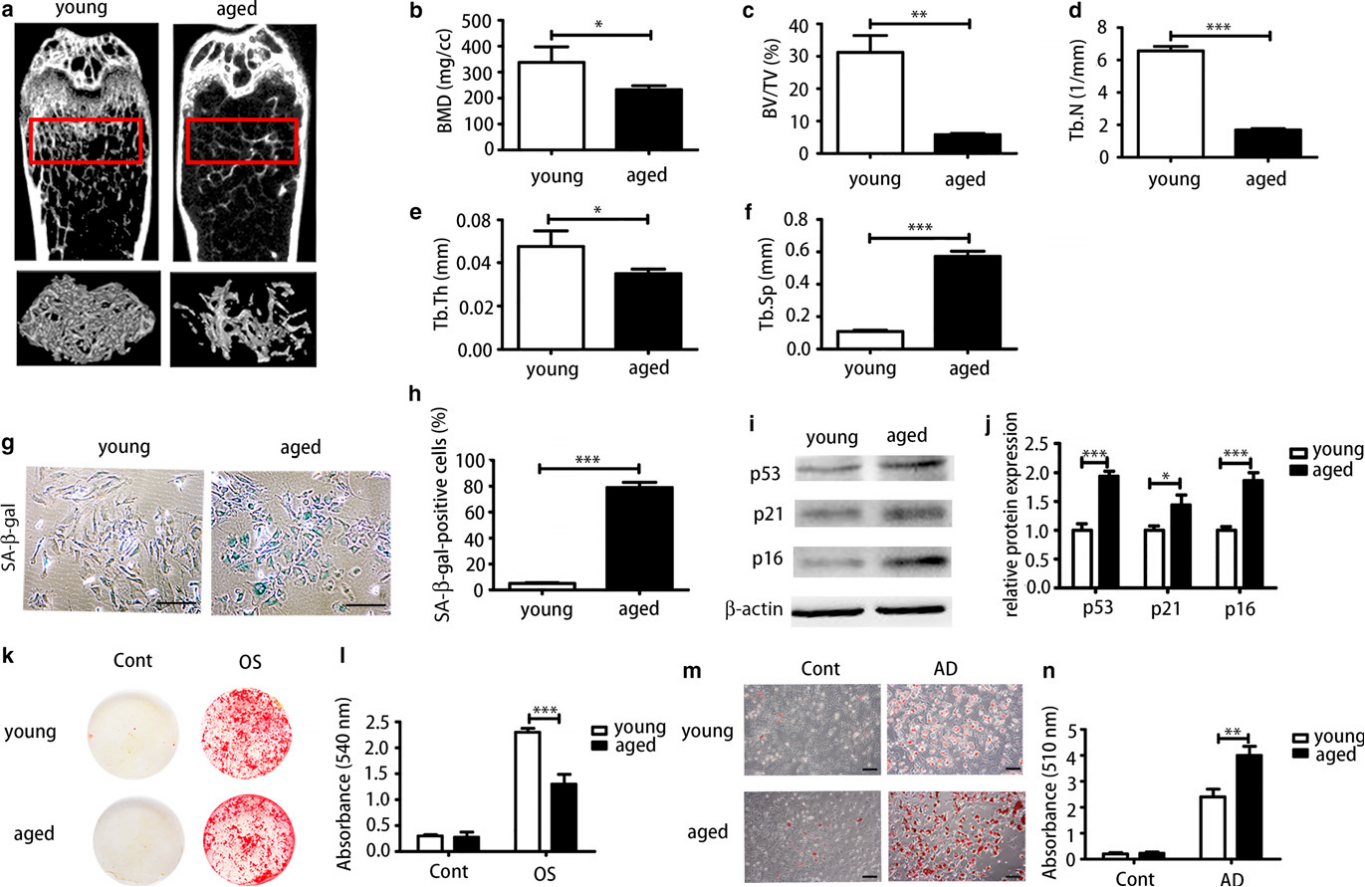
Aged bone marrow‐derived mesenchymal stem cells (BMMSCs) showed degenerative properties in senile osteoporosis compared with young BMMSCs. (a) Micro‐CT analysis of trabecular bone mass in the femora of 3‐month‐old (young) and 16‐month‐old (aged) mice. Quantitative analysis was performed including (b) bone mineral density (BMD) (*N* = 3), (c) trabecular bone volume (BV/TV) (*N* = 3), (d) trabecular number (Tb.N) (*N* = 3), (e) trabecular thickness (Tb.Th) (*N* = 3) and (f) trabecular space (Tb.Sp) (*N* = 3). At the cellular level: (g, h) senescence‐associated β‐galactosidase analysis of cultured BMMSCs derived from young and aged mice and quantitative analysis of positive cells. Scale bar = 100 μm. (i, j) The protein level of senescence markers (p53, p21, p16) of young and aged cells were determined by Western blot. (k, l) Alizarin Red staining of young and aged BMMSCs. OS = osteogenically induced. (m, n) Oil Red staining of young BMMSCs and aged BMMSCs. AD = adipogenically induced. Scale bar = 100 μm. Results are presented as means ± *SD*. *n* = 3. *p < .05, **p < .01, ***p < .001

To assess bone mass with advancing age, the bone mass of femora of young (3, 3‐month‐old) and aged (3, 16‐month‐old) mice was analysed by micro‐CT (Figure 1a). The results revealed that bone mineral density (BMD) (Figure 1b) was decreased in aged femora compared with young femora; the effects included significantly reduced trabecular bone volume (BV/TV) (Figure 1c), trabecular number (Tb.N) (Figure 1d), trabecular thickness (Tb. Th) (Figure 1e) and increased trabecular space (Tb.Sp) (Figure 1f). Bone marrow‐derived mesenchymal stem cells from young and aged femora were harvested and cultured for analysis of cell biological properties. Bone marrow‐derived mesenchymal stem cells were identified by high expression of Sca‐1 and CD29 and simultaneous absence of CD34 and CD45. Senescence‐associated β‐galactosidase analysis (SA‐β‐gal) revealed that aged BMMSCs exhibited more β‐galactosidase‐positive cells (Figure 1g,h). Senescence‐associated markers (p53, p21, p16) of aged BMMSCs were significantly increased at protein level (Figure 1i,j). The osteogenic differentiation capacity of aged BMMSCs was significantly decreased compared with those obtained from young mice; this was evident from Alizarin Red staining (Figure 1k,l). Oil Red staining showed that aged BMMSCs possessed higher adipogenic differentiation capacity compared with young cells (Figure 1m,n). In addition, the self‐renewal capacity of aged BMMSCs was significantly reduced than young cells as indicated by colony‐forming unit (CFU) analysis (Figure S1).

### 2.2 Autophagy level declines in BMMSCs and bone with age

**Figure 2:**
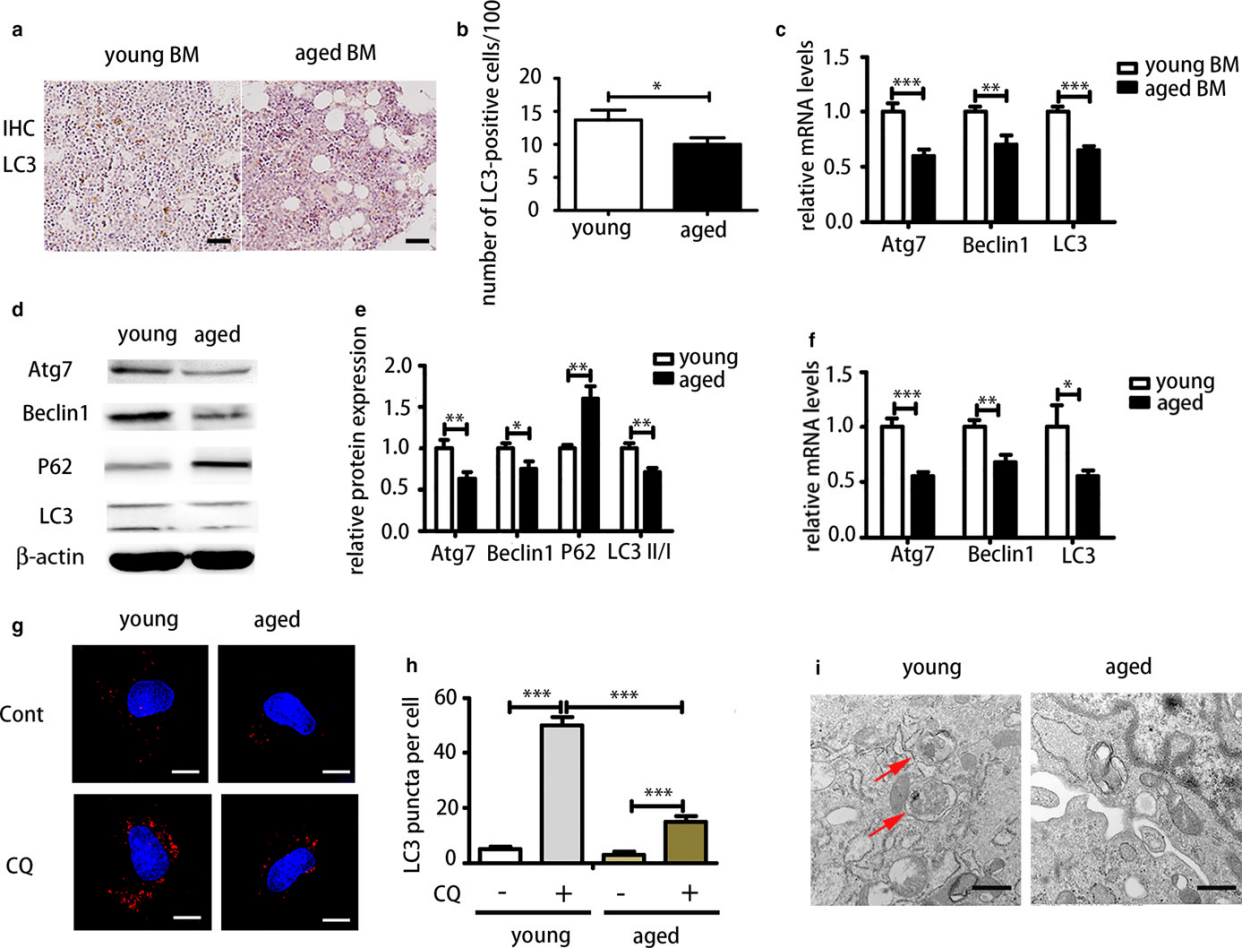
Comparison of autophagy of young and aged bone marrow and bone marrow‐derived mesenchymal stem cells (BMMSCs) showed that autophagy was decreased with aging. (a, b) LC3 of immunohistochemistry (IHC) of young and aged bone marrow. Scale bar = 200 μm. (c) Real‐time PCR analysis on whole bone marrow of young and aged femora. BM = bone marrow. (d, e) Western blot was performed to examine expressions of Atg7, Beclin1, P62 and LC3 in young and aged BMMSCs at protein level. (f) Real‐time PCR was performed to detect mRNA expression of Atg7, Beclin1 and LC3. (g, h) Immunofluorescence (IF) staining of LC3 in young and aged BMMSCs treated with CQ and PBS. Scale bar = 10 μm. (i) Transmission electron microscopy (TEM) was used to detect autophagosomes of young and aged BMMSCs. Scale bar = 500 nm. Results are presented as means ± *SD*. *n* = 3. *p < .05, **p < .01, ***p < .001

To compare autophagy levels of young and aged bone, we performed immunohistochemical (IHC) analysis and real‐time PCR on bone marrow. Immunohistochemical analysis of LC3 showed that both groups displayed positive expression of LC3 and aged bone marrow exhibited less positive staining than young bone marrow (Figure 2a,b). Accordingly, mRNA expressions of Beclin1, Atg7 and LC3 of aged bone marrow were significantly reduced compared with that of the young group (Figure 2c). Although from the results above we can tell autophagy‐associated genes and protein in aged bone marrow is reduced compared with that of young bone marrow, it is hard to tell which kind of cells contribute to this change mostly. To detect autophagy of BMMSCs specifically, different methods were applied to examine autophagy levels of young and aged BMMSCs. We analysed protein expression of key autophagy‐associated proteins Atg7, Beclin1, P62 and LC3II/I in young and aged BMMSC by Western blot. Significant reduction in protein expression of autophagy‐associated proteins Atg7, Beclin1 and LC3II/I and upregulation of P62 protein were observed in aged BMMSCs compared with that of young cells (Figure 2d,e). Real‐time PCR showed that autophagy‐associated genes were reduced in aged cells compared with that of young BMMSCs (Figure 2f). We then investigated autophagy flux in both young and aged BMMSCs by treating the cells with either vehicle or chloroquine (CQ), and analysing the levels of LC3 puncta by immunofluorescent assay. We found that young BMMSCs treated with either vehicle or CQ accumulate more LC3 dots compared with aged BMMSCs (Figure 2g,h). Autophagosomes could be identified by transmission electron microscope (TEM), and aged cells possess fewer autophagosomes than young cells (Figure 2i). Autophagosomes are spherical structures with double‐layer membranes that contain cytoplasmic material and/or organelles. Taken together, these data revealed that with aging, autophagy displayed a decreased tendency in both bone marrow and BMMSCs which might contribute to degenerative changes of bone and BMMSCs. Further investigation of the relationship between autophagy and degenerative changes of BMMSCs and bone is required to elucidate the possible mechanism.

### 2.3 Downregulation of autophagy by 3‐MA caused senescence of young BMMSCs

**Figure 3:**
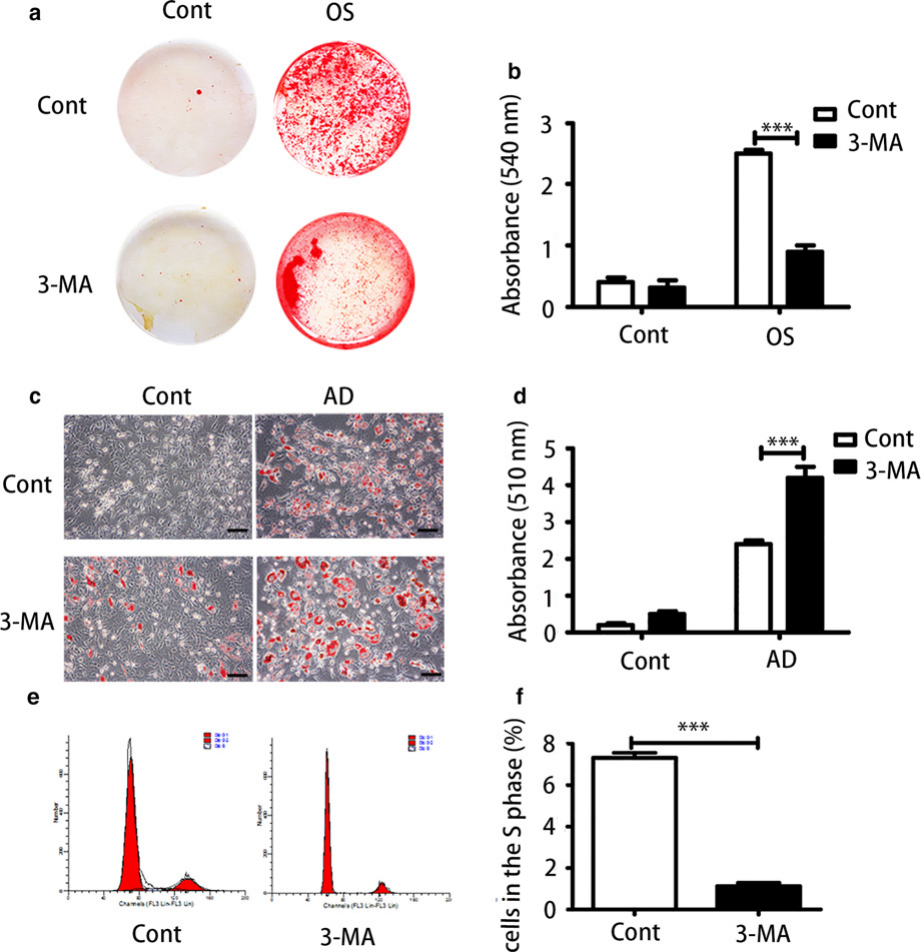
Inhibition of autophagy reduced proliferation and caused imbalanced differentiation of young bone marrow‐derived mesenchymal stem cells (BMMSCs). (a, b) Alizarin Red staining and quantitative analysis of young BMMSCs and 3‐MA‐treated young BMMSCs. (c, d) Oil Red staining and quantitative analysis of young BMMSCs and 3‐MA‐treated BMMSCs. Scale bar = 100 μm. (e, f) Cell proliferation capacity was evaluated by cell cycle analysis. Results are presented as means ± *SD*. *n* = 3. *p < .05, **p < .01, ***p < .001

As we observed that autophagy was significantly decreased in aged BMMSCs compared with young BMMSCs, we next investigated the relationship between autophagy and stem cell aging by manipulating autophagy levels of young and aged BMMSCs. The autophagy inhibitor 3‐MA was used in young cells to examine changes of osteogenesis, adipogenesis and proliferation. A concentration of 5 mm recommended in the literature could effectively reduce autophagy (Figure S2a,b). Alizarin Red staining showed that 3‐MA could significantly inhibit the osteogenic differentiation capacity of young BMMSCs (Figure 3a,b). As we had already observed that aged BMMSCs exhibited higher adipogenic differentiation capacity, Oil Red staining revealed that 3‐MA could significantly increase adipogenic differentiation capacity of young BMMSCs (Figure 3c,d). Cell cycle analysis showed that 3‐MA inhibited proliferation of young BMMSCs (Figure 3e,f). Overall, these results indicated that inhibition of autophagy by 3‐MA turned young BMMSCs into an aged state with decreased osteogenic differentiation capacity, increased adipogenic differentiation capacity and reduced proliferation, which suggested that autophagy might be a key regulator of stem cell senescence. However, further investigation of autophagy in aged BMMSCs is needed.

### 2.4 Upregulation of autophagy by rapamycin restored degenerative function of aged BMMSCs

**Figure 4:**
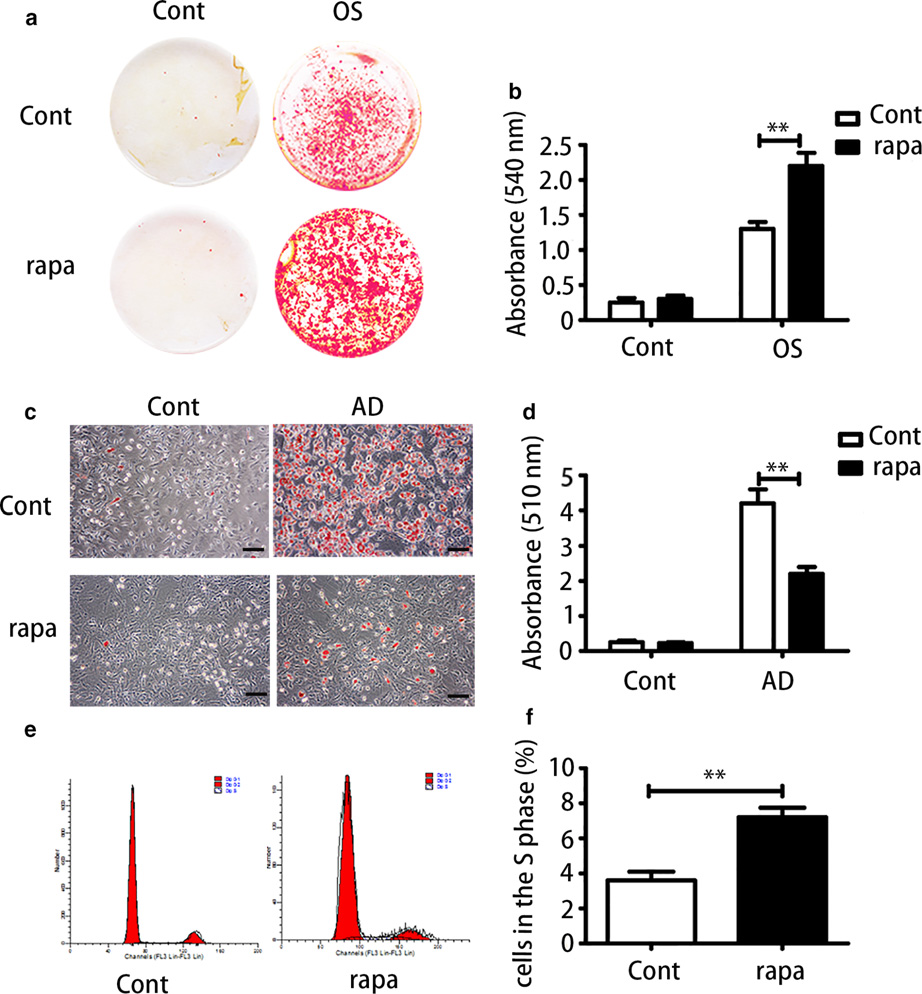
Rapamycin as an autophagy inducer could restore biological properties of aged bone marrow‐derived mesenchymal stem cells (BMMSCs). (a, b) Alizarin Red staining and quantitative analysis of aged BMMSCs and rapamycin‐treated aged BMMSCs. (c, d) Oil Red analysis and quantitative analysis of aged BMMSCs and rapamycin‐treated BMMSCs. Scale bar = 100 μm. (e, f) Cell cycle analysis of aged BMMSCs and rapamycin‐treated aged BMMSCs. Results are presented as means ± *SD*. *n *= 3. *p < .05, **p < .01, ***p < .001

Next, we used rapamycin as an inducer of autophagy to examine its effect on aged BMMSCs. A dose of 100 nm of rapamycin resulted in the greatest increase in autophagy levels (Figure S2c‐e). As shown previously, aged BMMSCs exhibited lower osteogenic differentiation capacity; we found that rapamycin could restore the osteogenic differentiation capacity of aged cells (Figure 4a,b). Meanwhile, rapamycin significantly reduced the adipogenic differentiation capacity of aged BMMSCs as shown by Oil Red staining (Figure 4c,d). Furthermore, rapamycin promoted proliferation of aged BMMSCs (Figure 4e,f). In conclusion, activation of autophagy by rapamycin could restore aged BMMSCs functions of differentiation and proliferation.

### 2.5 Autophagy regulated ROS and p53

**Figure 5:**
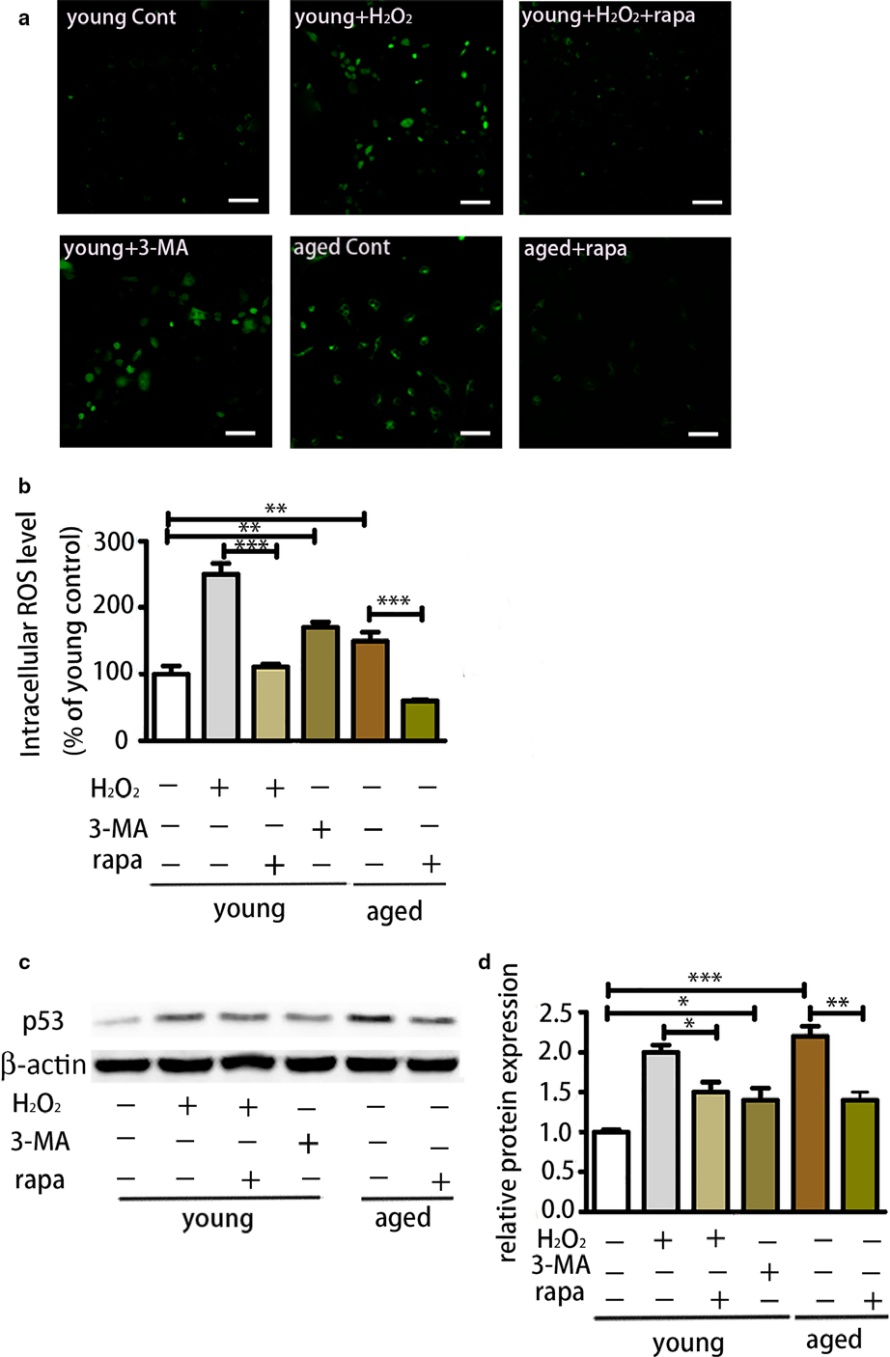
Activation of autophagy could reduce reactive oxygen species (ROS) levels and p53 expression, while 3‐MA reversed these effects. (a) ROS assessment of different groups of BMMSCs (young bone marrow‐derived mesenchymal stem cells [BMMSCs], H_2_O_2_‐treated young BMMSCs, H_2_O_2_‐ and rapamycin‐treated young BMMSCs, 3‐MA‐treated young BMMSCs and aged BMMSCs and rapamycin‐treated aged BMMSCs) by confocal microscopy. Scale bar = 100 μm. (b) ROS assessment of each group of cells by flow cytometry. (c, d) The protein level of p53 of each group of cells. Results are presented as means ± *SD*. *n *= 3. *p < .05, **p < .01, ***p < .001

We observed that activation of autophagy could alleviate aging of BMMSCs and restore aged BMMSCs' osteogenic differentiation and proliferation, while inhibition of autophagy could cause senescence of young BMMSCs. However, the underlying mechanism remained unclear. Therefore, we explored the possible mechanisms involved. Intracellular ROS levels of BMMSCs were detected based on primary fluorescence. First, we investigated ROS levels of young BMMSCs, H_2_O_2_‐treated young BMMSCs (100 μm), H_2_O_2_ (100 μm)‐ and rapamycin (100 nm)‐treated young BMMSCs, 3‐MA (5 mm)‐treated young BMMSCs and aged BMMSCs and rapamycin (100 nm)‐treated aged BMMSCs. The results showed that aged BMMSCs had higher ROS levels than young BMMSCs. Rapamycin could reduce ROS levels in both H_2_O_2_‐treated young BMMSCs and aged BMMSCs, while 3‐MA could induce more ROS in young BMMSCs (Figure 5a,b). Accordingly, protein levels of p53 had the same trend as ROS, which was that H_2_O_2_ increased expression of p53, while rapamycin could effectively reduce the expression of p53 in H_2_O_2_‐treated cells and aged cells. 3‐MA increased p53 expression of young BMMSCs. Therefore, autophagy might regulate BMMSC aging via the ROS and p53 pathways (Figure 5c,d). From these results, we concluded that activation of autophagy could reduce ROS levels and p53 expression, while inhibition of autophagy could increase ROS levels and p53 expression, causing senescence.

### 2.6 Autophagy activator restored bone loss in aged mice

**Figure 6:**
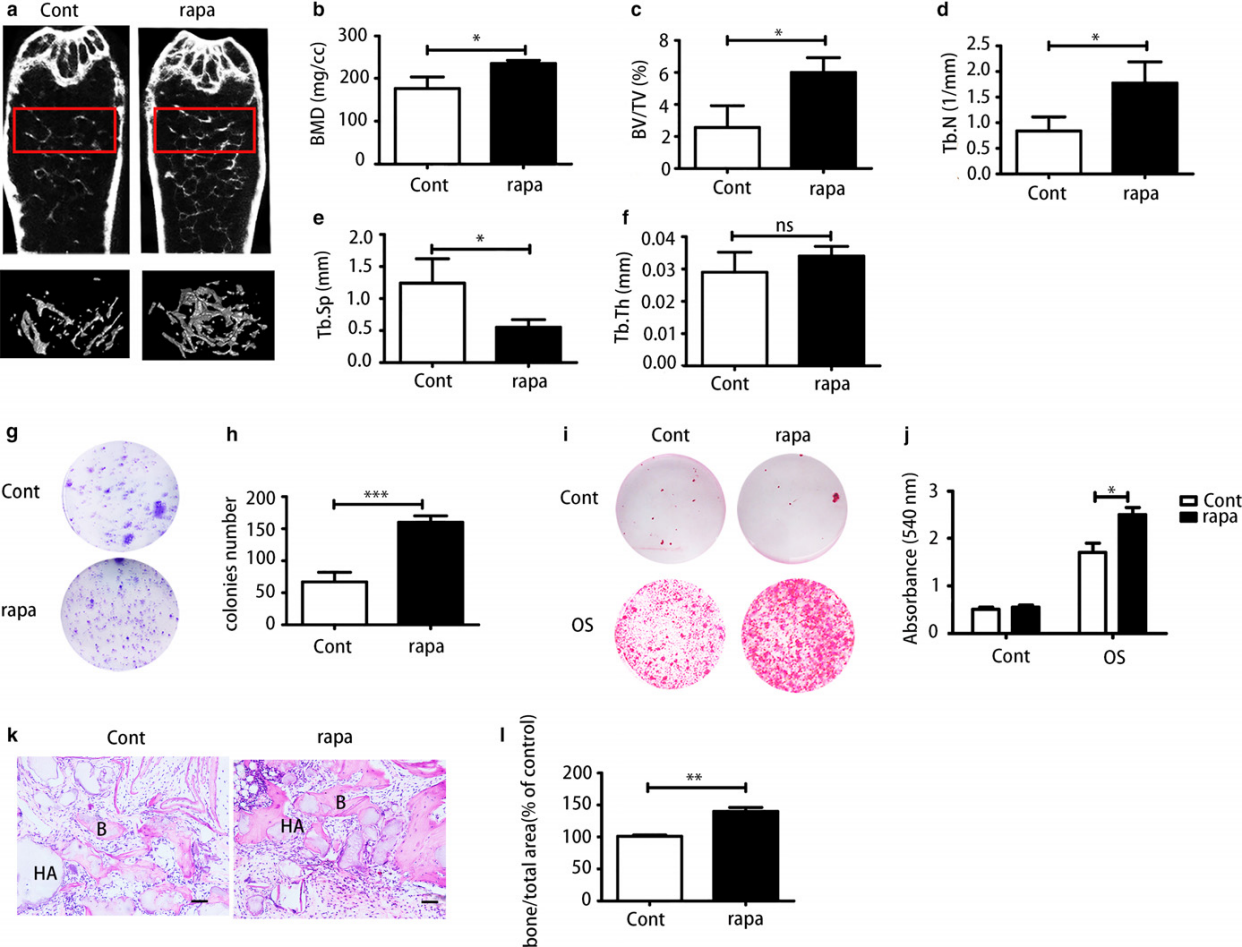
Rapamycin could restore the bone loss in aged mice. (a) Micro‐CT analysis of trabecular bone mass in the femora of the control group and rapamycin‐treated group. Quantitative analysis including (b) bone mineral density (BMD) (*N* = 3), (c) trabecular bone volume (BV/TV) (*N* = 3), (d) trabecular number (Tb.N) (*N* = 3), (e) trabecular space (Tb.Sp) (*N* = 3) and (f) trabecular thickness (Tb. Th) (*N* = 3). (g, h) CFU staining and quantitative analysis of bone marrow‐derived mesenchymal stem cells (BMMSCs) from both groups. (i, j) Alizarin Red staining and quantitative analysis of BMMSCs from the control group and rapamycin‐treated group. (k, l) H&E staining was used to determine BMMSCs osteogenesis capacity in nude mice. Scale bar = 100 μm. Results are presented as means ± *SD*. *n *= 3. *p < .05, **p < .01, ***p < .001

To determine whether autophagy plays a role in vivo, we used rapamycin to induce autophagy in aged male mice with senile osteoporosis. Micro‐CT analysis was carried out to examine the bone mass of rapamycin‐treated mice and DMSO‐treated mice. The analysis revealed that rapamycin restored bone mass of aged mice (Figure 6a); the effects included increased BMD (Figure 6b), BV/TV (Figure 6c), Th.N (Figure 6d) and reduced Tb.Sp (Figure 6e), while Tb.Th (Figure 6f) was not significantly changed. Bone marrow‐derived mesenchymal stem cells of primary culture from both groups were analysed. Western blot was used to detect autophagy levels of BMMSCs from both groups; rapamycin‐treated BMMSCs exhibited higher autophagy level with higher expression of Beclin1, LC3II/I (Figure S3). Colony‐forming unit analysis was carried out to determine the self‐renewal capacity; the rapamycin‐treated group produced more colonies than the control group (Figure 6g,h). Alizarin Red staining was performed after osteogenic induction for 14 days. Bone marrow‐derived mesenchymal stem cells from the rapamycin group exhibited higher osteogenic differentiation capacity than those from the control group (Figure 6i,j). Meanwhile, to further study whether autophagy enhances ectopic bone formation in vivo, BMMSCs from these two groups were combined with a hydroxyapatite–tricalcium phosphate scaffold (HA/TCP) and then implanted subcutaneously in NOD/SCID mice. After 8 weeks, BMMSCs from rapamycin‐treated mice produced more bone‐like tissue compared with those from the control group (Figure 6k,l). These data suggested that rapamycin could restore the bone loss in aged mice, which indicated that activation of autophagy could alleviate bone aging in mice. This might be a future target for clinical treatment of senile osteoporosis.

## 3 DISCUSSION

Various animal models have been established to investigate pathophysiology of osteoporosis and delineate mechanisms underlying bone loss. In C57BL/6J mice, patterns of age‐related changes in trabecular bone are quite similar to humans. Mice older than twelve months showed dramatic bone decrease especially (Glatt, Canalis, Stadmeyer, & Bouxsein, 2007). Moreover, bone loss in females is more associated with oestrogen. Thus, here we chose sixteen‐month‐old male C57BL/6J mice for study of senile osteoporosis.

Bone marrow‐derived mesenchymal stem cells have been regarded as the main source of osteoblasts for skeletal repair (Fibbe, 2002). It has been reported that degenerative changes of BMMSCs in humans and rodents during aging are associated with bone aging. Bone marrow‐derived mesenchymal stem cells tend to partially lose their self‐renewal capacity and differentiate into adipocytes instead of osteocytes with aging, which causes bone loss and fat accumulation (Moerman, Teng, Lipschitz, & Lecka‐Czernik, 2004). These changes include impaired capacity of proliferation, imbalanced differentiation and increased senescence (Sethe, Scutt, & Stolzing, 2006; Stenderup, Justesen, Clausen, & Kassem, 2003). Our findings showed that aged BMMSCs had decreased osteogenesis, elevated adipogenesis and decreased proliferation compared with young BMMSCs; these results are in line with the previous findings. Additionally, the p53/p21 pathway has been elucidated as a major regulator of aging. As tumour suppressor and cell cycle inhibitor, p16 is a marker of aging that accumulates in aged tissue (Janzen et al., 2006; Zindy, Quelle, Roussel, & Sherr, 1997). According to our study, both p53/p21 and p16 are activated in aged BMMSCs, which indicate that p53/p21 and p16 are involved in BMMSC aging. However, the underlying mechanism of BMMSC aging is still unclear and requires more precise elucidation.

Autophagy not only works as a simple cellular degradation tool for large molecules such as misfolded and aggregated proteins but is also involved in the removal of dysfunctional organelles such as mitochondria to maintain cellular metabolism (He & Klionsky, 2009). The predominant view of autophagy's role in aging is that autophagy is an effective anti‐aging mechanism that involves different pathways including the (mammalian) target of rapamycin (mTOR), insulin‐like growth factor binding to insulin‐like growth factor receptors (IGF1R), adenosine monophosphate‐activated protein kinase (AMPK) and p53 (Rubinsztein et al., 2011). Upregulation of autophagy has been reported to be able to extend the lifespan of aged mice and elder flies and restore the self‐renewal capacity of stem cells, which indicates that the anti‐aging effect is partially associated with restoring stem cell functions (Harrison et al., 2009; Simonsen et al., 2008). Here, we demonstrated that autophagy levels of aged BMMSCs were decreased compared with young BMMSCs. Numerous studies have indicated that with aging, autophagy decreases in different kinds of tissue such as kidney and brain (Kume et al., 2010; Lipinski et al., 2010). There are also studies revealing that autophagy is activated during aging in cells such as fibroblasts (Demirovic, Nizard, & Rattan, 2015) as in aged BMMSCs (Zheng et al., 2014). We demonstrated that autophagy is reduced in aged BMMSCs, which is consistent with the mainstream view. Thus, we speculate that decreased autophagy in aged BMMSCs might be one of the causes of degenerative changes of aged BMMSCs, and bone loss by decreased autophagy could be a potential new mechanism of bone aging.

The results of the manipulation of autophagy in both young BMMSCs and aged BMMSCs confirmed our speculations. As an autophagy inhibitor, 3‐MA was used on young BMMSCs; the results showed that inhibition of autophagy not only reduced osteogenesis and promoted adipogenesis but also inhibited proliferation of young BMMSCs, which indicated that decreased autophagy could turn young cells into an aged state with degenerative properties. Meanwhile, the autophagy inducer rapamycin could partially convert aged BMMSCs to a young state by increasing osteogenesis, reducing adipogenesis and promoting proliferation. Previous studies have shown that depletion of Atg7 of osteocytes mimics skeletal aging and defects of autophagy in muscle cells and T cells; this may lead to ROS accumulation, which may cause cellular damage (Masiero et al., 2009; Pua, Guo, Komatsu, & He, 2009). Accordingly, we observed elevated ROS and p53 protein expression in the 3‐MA‐treated group and reduced ROS and p53 expression in the rapamycin‐treated group; this result provides a molecular link to the age‐related changes in BMMSCs and autophagy. Reactive oxygen species has been widely reported as an essential marker and regulator of aging, causing molecular damage by regulating different pathways. Numerous reports have demonstrated that p53 is a crucial regulator of senescence and plays a dual role in tumour progression and suppression (Coppe, Desprez, Krtolica, & Campisi, 2010). Additionally, cell cycle arrest has been proven to be induced by p53 in the aging process (Vigneron & Vousden, 2010), which indicates that the cell cycle changes by autophagy activators and inhibitors that we observed above could be related to the p53 level. In human fibroblasts, autophagy regulates ROS‐induced senescence by p21 in a p38 MAPKα‐dependent manner (Luo et al., 2011). Also autophagy has been proven to be a crucial stem cell fate regulator via regulating ROS and DNA damage markers associated with p16INK4a and pS6 induction (Garcia‐Prat et al., 2016). Thus, the complex molecular network needs to be further explored.

Previous report found that rapamycin promotes osteoblast differentiation and inhibition of autophagosome formation in a mouse model showed severe osteopenia (Darcy et al., 2012; Shimada, Greer, McMahon, Bouxsein, & Schipani, 2008). Similarly, rapamycin has been proven to be able to reduce the severity of age‐related bone changes in trabecular bone of older male rats by partially activating autophagy of osteocytes (Luo, Ren, Li, Lian, & Lin, 2016), which is consistent with our findings. Also, rapamycin has been reported to be able to inhibit osteoclast number and formation in experimental animal models and reduce bone resorption in patients (Hocking, Whitehouse, & Helfrich, 2012). Therefore, the beneficial effects of rapamycin might be coordinated by different bone cells. In our study, the increase in bone mass in rapamycin‐treated mice is minor, albeit significant, as rapamycin was only applied in animals for 2 months at 1.5 mg/kg. Trabecular thickness was not increased significantly. Thus, increased treatment time and dose might be needed to improve the beneficial effects. Previous studies have observed that inhibition of mTOR1 prevents stem cell exhaustion and promotes stem cell function in vivo (Castilho, Squarize, Chodosh, Williams, & Gutkind, 2009), and our findings confirmed this conclusion. However, as a key regulator, the mTOR pathway plays various roles in most major cellular functions by regulating multiple pathways including S6K1, 4E‐BP1 and autophagy (Laplante & Sabatini, 2012). Thus, there also might be other mechanisms in addition to autophagy involved. Therefore, further studies concerning the details of the underlying mechanisms are needed.

There have been a few drugs applied clinically for the treatment of osteoporosis. Bisphosphonates and denosumab have been proved to be able to reduce the risk for vertebral, hip and nonvertebral fractures by suppressing osteoclastogenesis and/or osteoclastic activity. However, they have also been associated with a higher risk of adverse events such as osteonecrosis. Teriparatide, a parathyroid hormone analogue, can increase trabecular bone mass but has side effects such as concurrent stimulation of bone resorption and potential hypercalcemia (Dede, Makras, & Anastasilakis, 2017; Fukumoto & Matsumoto, 2017). Here, we proved that activation of autophagy might be a new avenue for treatment of osteoporosis and more autophagy inducer shall be considered in the future such as rapamycin, spermidine, lithium.

We demonstrated that autophagy was downregulated in aged BMMSCs and that autophagy played a vital role in maintaining the properties of BMMSCs. However, there are still some unanswered questions related to autophagy in senile osteoporosis. We had already known that there are sex differences in senile osteoporosis that possess different forms of pathogenesis and different mechanisms. The animal model we used here was male mice and thus could not represent the female postmenopausal model that is closely associated with oestrogen levels; our conclusions thus do not apply to females, and more studies need to be conducted on postmenopausal osteoporosis models.

In summary, based on our findings above, we conclude that activation of autophagy can restore degenerative properties of aged BMMSCs via regulating ROS level and p53 expression. Therefore, a new and promising target for amelioration of senile osteoporosis was elucidated, and this could be a potential avenue for the prevention of osteoporosis and treatment of bone aging.

## 4 MATERIALS AND METHODS

### 4.1 Animals

All animal procedures were approved by the Intramural Animal Use and Care Committee of Fourth Military Medical University, and all operations on animals followed the guidelines of Intramural Animal Use and Care Committee of the Fourth Military Medical University, Xi'an, China. Two‐month‐old C57BL/6J male mice were purchased from Animal Experimental Center of Fourth Military Medical University (Xi'an China). Twelve‐month‐old C57BL/6J male mice were purchased from Guangdong Medical Laboratory Animal Center (Guangzhou, Guangdong). All animals were housed under specific pathogen‐free conditions on a 12‐hr:12‐hr light–dark cycle with access to with free access to food pellets and tap water for up 6 months. Three‐month‐old and sixteen‐month‐old mice were used for experiments.

### 4.2 Micro‐computed tomography

Micro‐CT imaging was performed on the distal femora to analyse bone mass using Inveon micro‐CT (Siemens AG, Germany). X‐ray source was set at 80 kV, 500‐μA microfocus. We reconstructed image slices using micro‐CT image analysis software to produce three‐dimensional images. The region of interest was selected manually in the marrow cavity after threshold values were set. Ultra‐high‐resolution images of 11 μm of specimens were obtained. The relevant bone morphometric parameters, including BMD (mg/cc), bone volume relative to total volume (BV/TV, %), trabecular thickness (Tb. Th, mm), trabecular number (Tb. N, 1/mm) and trabecular spacing (Tb. Sp, mm) were assessed. The CT number for a Siemens phantom contained 4 bars of known densities was measured. The resulting Hounsfield unit (HU) values for each bar were then used to determine the HU‐to‐BMD conversion formula. BMD was calibrated as previously described according to the formula (Chityala, Pudipeddi, Arensten, & Hui, 2013).

### 4.3 BMMSC isolation and culture

Bone marrow‐derived mesenchymal stem cells were harvested from femora and tibiae of young C57BL/6J (3 months) and aged C57BL/6J (16 months) mice. Femora and tibiae were dissected out and cleaned of connective tissue. After clipping both ends of the bones, bone marrow was flushed out from femora and tibiae by a syringe to complete culture media that consisted of a‐MEM (Invitrogen, Carlsbad, CA, USA) supplemented with 20% foetal bovine serum (FBS, Atlanta Biologicals, Atlanta, GA, USA), 100 U/ml penicillin (Invitrogen), 100 μg/ml streptomycin (Invitrogen) and 12 μm l‐glutamine (Invitrogen). The cell suspension was then plated in 10‐cm culture dishes. The medium was changed every 2–3 days. After digestion with 0.25% trypsin/1 mm ethylenediaminetetraacetic acid (EDTA; Sigma‐Aldrich, St. Louis, MO, USA), cells were passaged at confluence.

### 4.4 Senescence‐associated β‐galactosidase staining

A total of 1 × 10^5^ BMMSCs per well were seeded in 12‐well plates and cultured for 3 days. β‐gal activity was assessed with a β‐gal staining kit (Beyotime Institute of Biotechnology, Jiangsu, China) according to the protocol provided. The numbers of senescent cells that were stained blue were counted, and the percentage was calculated.

### 4.5 Western blot

Cells were harvested and lysed with lysis buffer (Beyotime Institute of Biotechnology), and total protein concentration was assessed with BCA protein assay reagent (Bio‐Rad, CA, USA). Thirty micrograms of each sample was loaded and subjected to SDS‐polyacrylamide gels and then transferred to immunoblot PVD membranes (Millipore, Billerica, MA, USA). The membrane was blocked in blocking buffer (5% milk and 2 mg/ml BSA in PBST) for 1 hr and then incubated in primary antibodies against p53 (1:500; Cell Signaling), p21 (1:1,000; Cell Signaling), p16 (1:10,000; Cell Signaling), Atg7 (1:1,000; Cell Signaling), Beclin1 (1:1,000; Abcam), P62 (1:1,000; Abcam), LC3 (1:1,000; Abcam) and β‐actin (1:2,000; Abcam) in blocking solution overnight. After being washed in PBST for 10 min, membranes were incubated with secondary antibody in blocking solution for 2 hr at room temperature. After incubation, membranes were washed in PBST. An enhanced chemiluminescence kit (Amersham Biosciences, Piscataway, NJ, USA) was applied for visualization. ImageJ was used to quantify the results.

### 4.6 Real‐time PCR

Total mRNA was extracted from cells using TRIzol (Sigma‐Aldrich) reagent according to the provided protocol. Then, 2,000 ng mRNA was reverse‐transcribed with a PrimeScript RT reagent kit (Takara Bio Inc., Shiga, Japan). Settings of the program were 95°C for 10 min, 40 cycles of 95°C for 15 s and 60°C for 1 min. The primers for mRNA are listed below: Atg7 (F:ACCTCGCTGGGACTTGTGC;R:GGTGAATCCTTCTCGCTCGT), Beclin1 (F:CAGTACCAGCGGGAGTATAGTGA;R:TGTGGAAGGTGGCATTGAAGA), LC3 (F:CCTGTCCTGGATAAGACCAAGTT;R:CTCCTGTTCATAGATGTCAGCGAT), β‐actin (F:CTGGCACCACACCTTCTACA;R:GGTACGACCAGAGGCATACA).

### 4.7 Alizarin Red staining

Alizarin Red staining was applied for analysing osteogenesis. Cells were seeded at 50 cells/cm^2^ in 12‐well plates. After cells reached 80% confluence, osteogenic medium (a‐MEM supplemented with 20% FBS, 100 U/ml penicillin, 100 μg/ml streptomycin, 2 mm l‐glutamine, 2 mm β‐glycerol phosphate [Sigma‐Aldrich], 10 nm dexamethasone [Sigma‐Aldrich] and 100 μm l‐ascorbic acid 2‐phosphate [Sigma‐Aldrich]) was added and changed every 2–3 days. Rapamycin (Sigma‐Aldrich) and 3‐MA (Sigma‐Aldrich) were added in different experiments. After 14 days, cells were fixed in 4% formaldehyde at room temperature for 30 min and then stained with 0.1% Alizarin Red S (Sigma‐Aldrich) for 20 min at room temperature. To quantify the stain, we used 2% cetylpyridinium chloride (Sigma‐Aldrich) to elute the stain for 30 min and measured the spectrophotometric absorbance at 540 nm.

### 4.8 Oil Red staining

Oil Red staining was used for analysing adipogenesis. Cells were seeded at 50 cells/cm^2^ in 12‐well plates. After cells reached 100% confluence, adipogenic medium (a‐MEM supplemented with 20% FBS, 100 U/ml penicillin, 100 μg/ml streptomycin, 2 mm l‐glutamine, 5 μg/ml insulin [Sigma‐Aldrich], 50 μm indomethacin [Sigma‐Aldrich], 1 × 10^−6^ m dexamethasone and 0.5 μm 3‐isobutyl‐1‐methylxanthine [IBMX, Sigma‐Aldrich]) was added and changed every 2–3 days. Rapamycin and 3‐MA were added in different experiments. After 7 days, cells were fixed in 4% formaldehyde at room temperature for 30 min and then stained with 0.5% Oil Red (Sigma‐Aldrich) in methanol for 20 min at room temperature. To quantify the stain, we used methanol to elute the stain for 30 min and measured the spectrophotometric absorbance at 510 nm.

### 4.9 Immunohistochemical staining

Immunohistochemical staining was performed as described previously. Femora were dissected out from young and aged mice. Serial sections (9 μm) were obtained after samples had been fixed, decalcified and embedded. After deparaffinizing, rehydrating and antigen retrieval, sections were blocked in 10% normal serum with 1% BSA in TBS for 2 hr at room temperature followed by applying LC3 (1:200, Abcam) antibody to sections overnight at 4°C. After HRP polymer incubation for 30 min and development with chromogen for 10 min at room temperature, sections were dehydrated and mounted.

### 4.10 Transmission electron microscope

Both young and aged BMMSCs were harvested and fixed in 4% formaldehyde and 1% glutaraldehyde in 0.1 m PB (pH 7.4) overnight. After fixation, dehydration, embedding, sectioning and staining, samples were viewed with a TEM at an accelerating voltage of 80–120 kV.

### 4.11 Immunofluorescence analysis

Both young and aged cells were cultured on sterile coverslips and treated with 50 μM CQ (Sigma‐Aldrich) for 4 hr. The control group was treated with equal volumes of PBS. Cells grown on coverslips were fixed with 4% paraformaldehyde for 30 min and then permeabilized with 0.03% Triton X‐100 for 20 min. After that, 3% bovine serum albumin was used for blocking and then cells were incubated with LC3 (1:100, Abcam) antibody at 4°C overnight. After incubation with anti‐rabbit second antibody for 1 hr, nuclei were counterstained with Hochest 33258. Images were taken by a Zeiss LSM 510 laser‐scanning confocal microscope (Gottingen, Germany).

### 4.12 Cell cycle assessment

Young and aged BMMSCs were treated with 3‐MA and rapamycin, respectively, for 24 hr. The control group was treated with equal volumes of PBS and DMSO, respectively. After 24 hr drug treatment, cells were harvested and then washed twice with cold PBS. After that, cells were fixed in cold 70% ethanol at 4°C overnight. Then, 400 μl of 50 μg/ml stock of PI (BD Biosciences, USA) and 50 μl of a 100 μg/ml stock of RNase (Sigma‐Aldrich) were added, and cells were incubated for 30 min at 4°C. Cells from each group were then analysed via flow cytometry. The percentages of cells in the G1, G2 and S phases were calculated by the BD FACS software.

### 4.13 Measurement of ROS

We performed both qualitative and quantitative assay of ROS levels on BMMSCs which were cultured on coverslips and in 25‐cm^2^ flasks, respectively. After being cultured for 24 hr, cells were treated accordingly for 8 hr as young BMMSCs, H_2_O_2_‐treated young BMMSCs (100 μm), H_2_O_2_ (100 μm)‐ and rapamycin (100 nm)‐treated young BMMSCs, 3‐MA (5 mm)‐treated young BMMSCs and aged BMMSCs and rapamycin (100 nm)‐treated aged BMMSCs. An ROS test kit (Genmed, Shanghai, China) was used to detect intracellular ROS level. For cells cultured on coverslips, DCFH‐DA (50 μm) was added and the cells were incubated for 30 min at 37°C. Then, the reaction solution was removed and fluorescence of cells was detected by confocal microscopy. For cells cultured in 25‐cm^2^ flasks, after being trypsinized and washed, the cells were incubated in DCFH‐DA (50 μm) for 30 min with gentle agitation. Then, the reaction was stopped, and flow cytometry with excitation at 488 nm was applied for quantitative analysis.

### 4.14 Rapamycin treatment in vivo

Sixteen‐month‐old male mice (*n* = 14) were randomly divided into two groups (7/each group) as a control group and a rapamycin group. In the rapamycin group, rapamycin was administered at 1.5 mg/kg every other day for 2 months through intraperitoneal injection. DMSO was injected in the control group. After 2 months of injections, mice from both groups were sacrificed and three femora from each group were collected for micro‐CT scanning. Bone marrow‐derived mesenchymal stem cells from femora and tibiae of the other mice were harvested and cultured for Western blot analysis, CFU analysis and Alizarin Red analysis. Bone marrow‐derived mesenchymal stem cells from these two groups were combined with a hydroxyapatite–tricalcium phosphate scaffold (HA/TCP) and then implanted into subcutaneous pockets on the backs of the 8‐week‐old NOD/SCID mice (Fourth Military Medical University). After 8 weeks, the implants were fixed with 4% paraformaldehyde and decalcified with 10% EDTA (pH 6.0). H&E staining was applied to detect histology.

### 4.15 CFU analysis

To assess the self‐renewal capacity of BMMSCs, 1 × 10^5^ primary cultured BMMSCs from control and experimental groups were seeded in 5‐cm dishes. After 10 days' culture, 4% paraformaldehyde was used to fix the cells, and then 0.1% toluidine blue was applied to stain the colonies. Colonies of more than 50 cells were counted and analysed.

### 4.16 Statistical analysis

Data are presented as mean ± *SD*. Comparisons were made using a *t* test and one‐way ANOVA for experiments with more than two groups. All experiments were repeated at least three times. *p* < .05 was considered significant.

## CONFLICT OF INTEREST

The authors declare no potential conflict of interests.

## AUTHORS' CONTRIBUTION

Yan Jin and Wenjia Liu designed the experiments. Yang Ma and Meng Qi did the majority of the experiments and collected data. Ying An analysed the data. Liqiang Zhang, Rui Yang and Daniel H Doro participated in the experiments. Yang Ma and Wenjia Liu drafted the manuscript.
