# Supplementary material for: Autophagy controls mesenchymal stem cell properties and senescence during bone aging
Source: Aging Cell. 2017 Dec 6;17(1):e12709. doi: 10.1111/acel.12709 (PMC5770781; doi:10.1111/acel.12709)
Supplement: Supplementary file 1 [file ACEL-17-na-s001.pdf]

Supplementary Fig.1

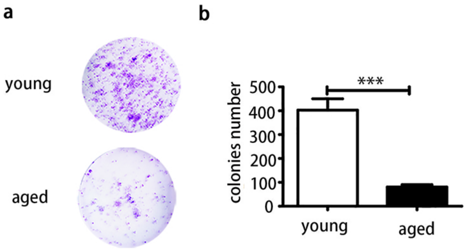

Aged BMMSCs presented reduced self-renewal capacity.
(a, b) CFU staining of young and aged BMMSCs.

Supplementary Fig.2

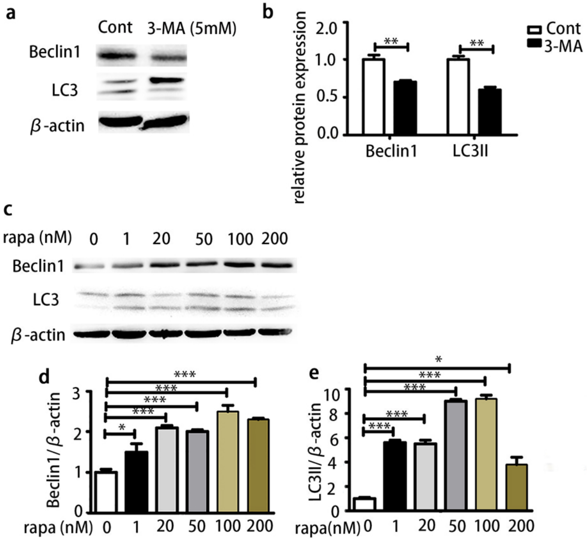

(a, b) 5mM 3-MA could reduce autophagy effectively.
(c-e) 100 nM rapamycin could increase autophagy mostly.

Supplementary Fig.3

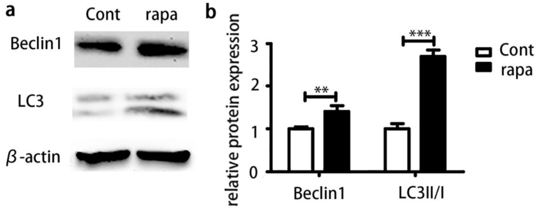

(a, b) BMMSCs from rapamycin-treated mice exhibited increased autophagy than control group by western blot.
